# The CaSR in Pathogenesis of Breast Cancer: A New Target for Early Stage Bone Metastases

**DOI:** 10.3389/fonc.2020.00069

**Published:** 2020-02-05

**Authors:** Souvik Das, Philippe Clézardin, Said Kamel, Michel Brazier, Romuald Mentaverri

**Affiliations:** ^1^MP3CV, EA7517, CURS, University of Picardie Jules Verne, Amiens, France; ^2^INSERM, Research Unit UMR_S1033, LyOS, Faculty of Medicine Lyon-Est, University of Lyon 1, Lyon, France; ^3^Department of Oncology and Metabolism, Medical School, University of Sheffield, Sheffield, United Kingdom; ^4^Department of Biochemistry, Amiens-Picardie University Hospital, Amiens, France; ^5^Faculty of Pharmacy, University of Picardie Jules Verne, Amiens, France

**Keywords:** CaSR, calcium-sensing receptor, breast cancer, mammary gland, bone metastasis, calcilytics

## Abstract

The Ca^2+^-sensing receptor (CaSR) is a class-C G protein-coupled receptor which plays a pivotal role in calciotropic processes, primarily in regulating parathyroid hormone secretion to maintain systemic calcium homeostasis. Among its non-calciotropic roles, where the CaSR sits at the intersection of myriad processes, it has steadily garnered attention as an oncogene or tumor suppressor in different organs. In maternal breast tissues the CaSR promotes lactation but in breast cancer it acts as an oncoprotein and has been shown to drive the pathogenesis of skeletal metastases from breast cancer. Even though research has made great strides in treating primary breast cancer, there is an unmet need when it comes to treatment of metastatic breast cancer. This review focuses on how the CaSR leads to the pathogenesis of breast cancer by contrasting its role in healthy tissues and tumorigenesis, and by drawing brief parallels with the tissues where it has been implicated as an oncogene. A class of compounds called calcilytics, which are CaSR antagonists, have also been surveyed in the instances where they have been used to target the receptor in cancerous tissues and constitute a proof of principle for repurposing them. Current clinical therapies for treating bone metastases from breast cancer are limited to targeting osteoclasts and a deeper understanding of the CaSR signaling nexus in this context can bolster them or lead to novel therapeutic interventions.

## Introduction: The Calcium Sensing Receptor

The concept that extracellular Ca^2+^ acts directly on parathyroid cells to regulate PTH secretion had been afloat since the 1960's (1, 2). However, the first precise assertion of the hypothesis suggesting a “possible existence of a divalent cation receptor” on the cellular surface came from electrophysiological experiments performed in rat parathyroid cells in 1983 (3). The concept of a calcium receptor gained traction in the 1980's, and by 1990 several characteristics had been revealed. In 1991, two independent groups (4, 5) showed a viable strategy for cloning the calcium receptor in X*enopus* oocytes, an approach later used by Ed Brown et al. in cloning the cDNA encoding the bovine parathyroid calcium receptor (6). The irrefutable evidence on the existence of the receptor in 1993 was further reinforced by the clinically significant discovery that mutations in the calcium sensing receptor gene gave rise to inherited disorders of disrupted calcium homeostasis (7).

The extracellular CaSR is a dimeric class-C G protein-coupled receptor (GPCR), closely related to metabotropic glutamate receptors, gamma-aminobutyric acid type B (GABA_B_) receptors, various taste receptors and pheromone receptors. The human CaSR is a 1,078 amino acid protein, with a large 612 amino acid extracellular domain making up two lobes which adopt a Venus flytrap (VFT) conformation (8). Upon agonist stimulation, an open cleft of the VFT closes in, which is believed to induce conformational changes in the other domains, initiating signal transduction (9). Although the nomenclature points toward the main ligand of this receptor (Ca^2+^ ion), it does little to disclose its promiscuity of responding to various di- and trivalent cations, basic polypeptides, amyloid β-peptides and some aminoglycoside antibiotics (10–14). These constitute orthosteric agonists or type I calcimimetics which stimulate the receptor in the absence of Ca^2+^ or increases the sensitivity to calcium, albeit with different potencies. The second type of CaSR agonists are called allosteric modulators. These generally bind to a site different from that of orthosteric agonists, affecting the signaling and affinity of the orthosteric agonists either positively (calcimimetics) or negatively (calcilytics).

Signaling through the CaSR is multifaceted. Based on the majority of studies of this receptor in parathyroids, it has been shown to mainly interact with Gq/G11 heterotrimeric G protein (15, 16). Various intracellular cascades finally lead to a decrease in the secretion of parathyroid hormone (PTH) and a reduction in renal tubular Ca^2+^ reabsorption (17). Intracellular Ca^2+^ kinetics has been reported to be influenced by G12/13 pathways in different cell types. An example of such a modulation has been reported in the bone, where a G12/13 mediated activation promoted osteoblastic differentiation and downregulated osteoclastogenesis (18, 19). Also, since G12/13 signaling has been implicated in cell migration, it has been hypothesized to aid metastatic spread of breast and prostate tumors (20–22). CaSR mediated Gs signaling has been observed in pituitary cells and has been shown to affect human fetal lung development (23, 24). Being a multimodal chemosensor involved in transducing extracellular metabolic signals, the CaSR is also involved in preferential activation of distinct intracellular pathways in a phenomenon termed as “biased signaling” or “stimulus bias” (25). This is being leveraged in contemporary strategies for drugs targeting GPCRs (including the CaSR) while minimizing side-effects (26, 27). The alternation in coupling of G-proteins between normal and transformed breast cells was first hypothesized by Mamillapalli et al. and we have summarized it separately in our review as this is an important facet. This section aims to provide an opportunity to appreciate the various evidences of multiple G-protein couplings of this GPCR without deep-diving into the details of the downstream signaling pathways. For a comprehensive discourse on signaling, one can refer to an excellent review by Conigrave et al. (25).

The CaSR senses minor perturbations in serum Ca^2+^ levels and thus maintains an equilibrium by tightly regulating PTH secretion, renal calcium control, and bone remodeling. When the CaSR senses a dip in the extracellular Ca^2+^ concentration, it induces PTH secretion from the parathyroid glands. The secreted PTH acts by reducing kidney Ca^2+^ excretion, increasing intestinal Ca^2+^ absorption, and increasing bone resorption to release skeletal Ca^2+^. On the other hand, an increase in the physiological Ca^2+^ level causes receptor activation and inhibition in PTH synthesis and secretion (28). As already mentioned, the physiological significance became apparent when various inherited disorders like familial hypocalciuric hypercalcemia (FHH) and neonatal severe hyperparathyroidism (NSHPT) were found to be caused by loss-of-function mutations in the CaSR gene (7). Alternatively, gain-of-function mutations were found to give rise to autosomal dominant hypocalcemia (ADH) and Bartter Syndrome type V (29, 30). However, the receptor is not restricted to calcium homeostasis but also has been implicated in diverse processes like cellular proliferation, cellular differentiation, secretion, and gene expression in different tissues such as stomach, intestines, skin, brain, liver, and heart (31–39). Before delving into a rationale for targeting this versatile receptor in breast cancer, we will briefly look into its function in the mammary gland and how it plays a role in cancer progression.

## CaSR in the Normal Mammary Function

The first report of localization and confirmation of expression of CaSR in human breast tissues was published by Dr. Edward M. Brown's laboratory (40). They observed the expression of both mRNA and protein levels in ductal epithelial cells which was further confirmed by successive findings in mice (41). Immunofluorescence staining of lactating glands revealed the expression of the receptor in luminal epithelial cells at the basolateral surface and in the cytoplasm (41, 42). Although it is reasonable to surmise that the CaSR is mainly located on epithelial cells in the breast, these studies do not exclude minimal presence of the receptor in stromal cells (43). The role of the receptor was elucidated to be involved in controlling lactation by modulating the production of PTHrP and regulating calcium (41). The expression of CaSR in mammary epithelial cells was subsequently shown to be upregulated during lactation and downregulated during pregnancy (41). To circumvent neonatal deaths from a homozygous CaSR gene disruption, the CaSR gene was disrupted in mammary epithelial cells in mice at the onset of lactation which resulted in altered maternal calcium homeostasis (44). This genetic ablation had a domino effect- the lactating mothers were hypercalcemic, showed decreased PTH secretion (with increased renal excretion of calcium), increased secretion of PTHrP into milk, and reduced calcium transport into the milk (44). The suckling neonates demonstrated decreased calcium accumulation, evident from their ash calcium content (44). Although the lactating breast coordinates maternal and neonatal calcium homeostasis, it can be concluded from studies till date that the CaSR does not play a pivotal role in the development or differentiation in the normal mammary gland. The caveat of this conclusion is that most studies have focused on the period of lactation where there is the highest expression of CaSR (42).

Culmination of intensive work at understanding the pathophysiology of humoral hypercalcemia of malignancy (HHM) led to the discovery of the parathyroid hormone-related protein (PTHrP) (45). Owing to the N-terminal homology of PTH and PTHrP, both interacts with the same GPCR termed as type 1 PTH/PTHrP receptor (PTH1R). Unlike PTH which functions like a peptide hormone, PTHrP does not circulate (except during lactation and cancer) (46). In *Pthlh*^−/−^ and *Pth1r*^−/−^ embryos, loss of PTHrP signaling led to arrested mammary and nipple morphogenesis; while the overexpression of PTHrP (via the keratin 14 promoter) gave rise to ectopic nipples (45–47). A classic endocrine negative feedback loop is set up by CaSR signaling in the lactating breast which suppresses PTHrP production to counter bone calcium resorption, which has been proved both genetically and pharmacologically (41, 48).

## CaSR in Breast Cancer

**Figure 1:**
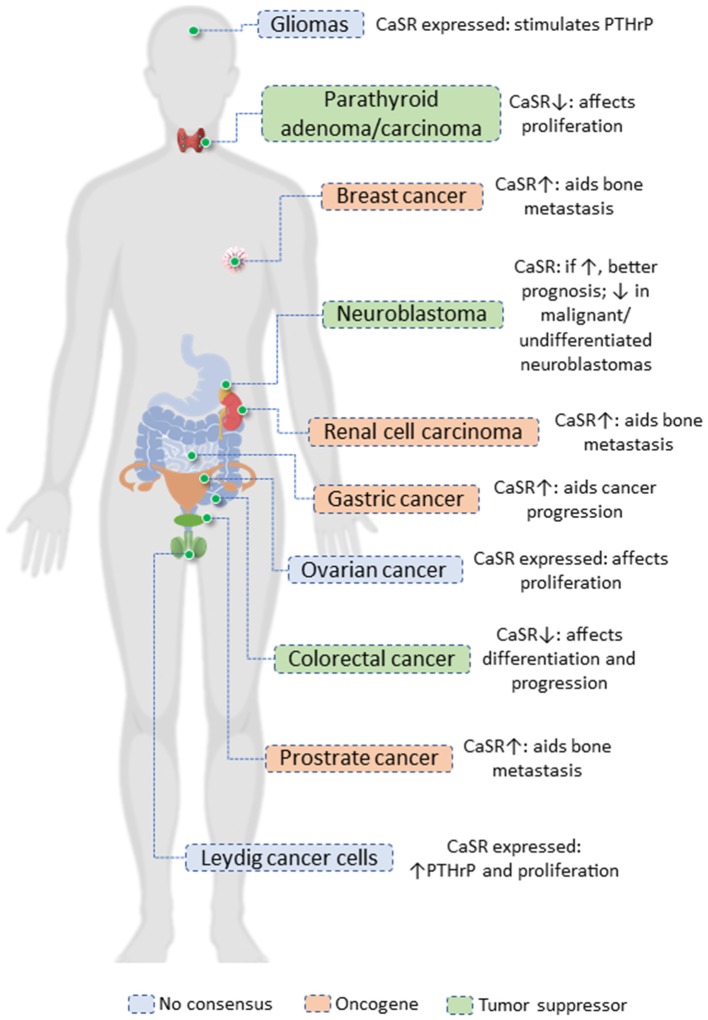
The role of CaSR in cancer.

The CaSR seems to function as an oncogene or tumor suppressor gene based on the cancer site (Figure 1). The expression of CaSR is greatly reduced or nullified in neuroblastomas, parathyroid cancer, and colorectal cancer (49–51). In our tissue of interest, the mammary gland, most of the evidences point toward its role in promoting cancer progression. Besides this, the CaSR also acts as an oncogene in several cancers such as ovarian, prostate, and testicular cancer (52–54). Although we will be focusing on the mammary gland, it is important to keep in mind the tissues where the CaSR aids tumor progression; cumulative evidences of similar function in different tissues would help us decipher the intricacies of the signaling aspects of the CaSR.

### Genetic Aspects of the CaSR

Studies have aimed to find a correlation between breast cancer risk and single nucleotide polymorphisms (SNPs) of the CaSR gene. According to a recent review, only four articles (comprising of case-control and meta-analyzes studies) point toward a significant association (55). The African-American Breast Cancer Epidemiology and Risk (AMBER) study demonstrated that the SNP in CASR rs112594756 presented with a higher odds ratio for estrogen receptor status in breast cancer (56). Li et al. found that the SNP rs17251221 was associated with reduced mRNA and protein levels of CaSR and could be a risk factor as well as a prognostic indicator of breast cancer. It is noteworthy that the same intronic polymorphism with “AG” and “GG” genotypes lowered the risk for ovarian cancer, even if it didn't correlate with survival (57, 58). CaSR SNP at rs1801725 was shown to have associated breast cancer with circulating calcium levels. Wang L. et al. showed that the decreased sensitivity of the CaSR to calcium due to the aforementioned polymorphism might predispose risk of breast cancer in up to 20% of cases with aggressive breast tumors linked to calcium in circulation (59). BRCA1 is a well-characterized tumor-suppressor gene, which is involved in various cellular functions and women who carry a mutated BRCA1 allele are at higher risk of developing breast cancer. Functional linkage studies between the CaSR and BRCA1 showed that cells harboring BRCA1 mutants with loss of expression of BRCA1 had downregulated CaSR expression. Data from these studies also showed that BRCA1 functioned through the CaSR in inhibiting survivin (an anti-apoptotic factor) expression. This means that the CaSR could rescue, in part, the deleterious effect of loss in BRCA1 function (60).

### PTHrP-CaSR Axis

We already discussed the role of PTHrP in the normal functioning of the breast, but it becomes interesting how it interacts with the CaSR in contributing to pathogenesis. Some excellent research by the Wysolmerski lab led to a possible explanation of the opposing effects of CaSR on PTHrP production based on alternate G-protein coupling. Generally, PTHrP secretion is suppressed by rising calcium levels in the normal breast tissue, but it is stimulated in breast cancer cells. It was seen that the CaSR used the Gα_i_ coupling in normal mammary epithelial cells but switched to Gα_s_ in MCF-7 and Comma-D cells. The alternative coupling stimulated adenyl cyclase activity (as opposed to inhibition in non-transformed cells), resulting in activation of cAMP/PKA pathways which are known to regulate PTHrP gene expression and PTHrP secretion (61). Manipulating the cAMP levels independent from CaSR activity was seen to produce a similar effect in PTHrP production in the mentioned cell types (62). Activation the CaSR can also act in a concerted way with transforming growth factor β to promote PTHrP secretion, as seen in MCF-7 and MDA-MB-231 cells (48).

PTHrP was discovered to be a causal factor in HHM but has been subjected to conflicting reports in its function in primary tumors. While some reports suggest that PTHrP production by primary breast tumors is a marker of bone metastases, others, notably a large prospective study, suggested that PTHrP production by the primary tumor confers a “less invasive phenotype” and inversely correlates with bone metastases (63, 64). The PTHrP gene, on the other hand, was recently identified as a genomic locus for breast cancer susceptibility (65). However, efforts aimed at deciphering the relation between CaSR and PTHrP in breast cancer demonstrated that the action of CaSR is mediated by nuclear PTHrP and partly affects proliferation and apoptosis. When a mutant form of PTHrP which couldn't translocate into the nucleus was transduced into cells, they failed to rescue the phenotype affected by depletion of the CaSR. When either the CaSR or PTHrP was knocked down in BT474 and MDA-MB-231 cells *in vitro*, it sensitized them to cellular death in response to increasing concentrations extracellular calcium. Furthermore, ablating the CaSR in MMTV-PyMT (mouse mammary tumor virus-polyoma middle tumor-antigen) transgenic mouse model tumor cells promoted apoptosis and inhibited growth *ex vivo* (66). Mice bearing C26-DCT colon tumors treated with Cinacalcet to reduce hypercalcemia presents an interesting case as the tumor cells do not express the CaSR. The attenuation in PTHrP-mediated increase in serum Ca^2+^, as observed in parathyroidectomized rats in which hypercalcemia had been induced with PTHrP injections, resulted from increased secretion of calcitonin and suggests calcimimetics didn't act directly on the tumor cells (67, 68).

## Bone Metastasis

Following Paget's seed and soil hypothesis, the bone microenvironment provides a fertile “soil” for breast and prostate cancer “seeding,” among other carcinomas, by hosting a plethora of biochemical or physical properties that makes it attractive for tumor growth (69, 70). This metastatic niche also provides homing signals like pH and extracellular Ca^2+^, which can be said to lure the cancer cells. Our team showed that highly bone-metastatic cells were prone to a greater migratory effect compared to less metastatic ones in response to Ca^2+^ and siRNA directed against the CaSR was able to mitigate that effect (71). Taking that hypothesis forward, we were also able to show *in vivo* that overexpression of a functional CaSR in MDA-MB-231 cells greatly increased their osteolytic potential mediated by epiregulin secretion, and downregulation of OPG (Osteoprotegerin) in osteoblastic cells, which could upregulate osteoclastogenesis (72). As adhesion also plays a key role in metastasis, Tharmalingam et al. reported for the first time that the coupling of CaSR and integrins in rat medullary thyroid carcinoma cells, along with release of [Ca^2+^]_i_, promotes adhesion and migration (73). This builds upon the studies which have shown that CaSR aids metastasis and hematopoietic stem cell harboring in adult bone marrow- which are also dependent on integrin-mediated cell adhesion (74, 75).

Before cancer cells find their way into the circulation, they may have to adopt an invasive phenotype, a phenomenon referred to as the epithelial–mesenchymal transition (EMT). During this adoption, an overlooked feature of breast tumors is the biological significance of microcalcifications *in situ*, mainly comprising of calcium oxalate or hydroxyapatite (76, 77). This had been partly explored long back, where the osteotropism of breast cancer was correlated with its ability of inducing microcalcifications by expressing bone matrix proteins (78). Clinically, mammographic calcifications aid in distinguishing benign from potentially malignant changes (79). Although calcium oxalate is mostly associated with benign tumors, hydroxyapatite has been linked to both benign and malignant ones (80). Taken together, it points toward a significant role of calcium signaling. Davis et al. found that the EGF-induced EMT in MDA-MB-468 cells was calcium signal dependent (81). Activation of the CaSR in breast cancer cells have also been shown to stimulate cell proliferation acting through membrane metalloproteinases, upregulating the transient receptor potential channel 1, stimulating EGFR, and ERK1/2 phosphorylation (82, 83). The link between CaSR and EMT in breast cancer is yet to be explored, but studies have shown that inducing CaSR in colon cancer (where it acts as a tumor-suppressor) inhibits EMT and lower expression in lung adenocarcinomas promotes a mesenchymal phenotype (84–86). The estrogen receptor alpha (ER) expression in primary breast cancers is linked to incidence of bone metastases and its activity is a clinically important metric (87). It was reported that high Ca^2+^ levels which are released during tumor induced bone resorption, and specific CaSR agonists increases ER transcriptional activity and decreases ER protein levels (88). Although there is a need for confirming an unequivocal role of the CaSR in bone metastasis, we can still speculate the mechanisms by which the bone microenvironment might act through the CaSR in promoting osteolysis or bone metastases. The tumor cells needs to adapt to this Ca^2+^ rich microenvironment to proliferate and promote osteolysis, and increased PTHrP secretion might be contributing to this by its paracrine actions (61). Bone marrow stromal cells and osteoblasts express the PTH1R which binds to PTHrP produced by skeletal metastatic breast cancer cells initiating the vicious cycle and is exacerbated by the calcium-CaSR signaling. Of all the factors released during bone resorption, matrix-derived growth factors can aid tumor cell survival and/or PTHrP production (43), feeding the cycle of osteolysis and thus, investigating the CaSR-PTHrP axis might open doors on a therapeutic front. If metastatic cell growth can be halted, the tumor-bone vicious cycle can be targeted, and the bone microenvironment can be rendered inhospitable for tumor colonization- it can be the ideal therapeutic option, which makes targeting the PTHrP-CaSR axis seems so compelling.

In the “vicious cycle” fueled by tumor cells that leads to a disrupted osteoblast-osteoclast coupling, the CaSR may also serve as a target on osteoclasts. It was shown that by either antagonizing the receptor on osteoclasts or in those lacking a functional CaSR led to increased apoptosis induced by high extracellular Ca^2+^ and also impaired osteoclastogenesis. Their data also suggested that CaSR mediated NF-κB translocation to the nucleus of osteoclasts and their activation led to apoptosis of mature osteoclasts (89). Identifying the interplay between the CaSR and various factors which aid tumor cell homing, survival and proliferation in the bone microenvironment can shed light on the extent it is involved in the processes.

## Calcilytics and Mode of Action

The definition of “calcilytics” is based on its function and doesn't account for its structure or CaSR-binding sites. The rationale for developing such compounds stemmed from the requirement of alternative small molecule calcilytics for treating osteoporosis; the standard of care at that time being anabolic therapies using PTH analogs (teriparatide) and PTH-related peptides (90). In 2001, the compound labeled as NPS-2143 became the first one to be reported in having the ability to inhibit CaSR activity (91, 92). Its potency was apparent, having an IC_50_ of 43nM in blocking Ca^2+^ accumulation in response to receptor activation (carried out in HEK 293 cells), going as high as 3 μM without affecting several other structurally similar GPCRs. Intravenous infusions in normal rats caused a rapid 4- to 5- fold increase in circulating PTH levels, and plasma Ca^2+^ levels were significantly elevated only after 90 min into the infusion with a slow return to baseline levels. Pharmacokinetic studies revealed an oral bioavailability of 11% in rats and high rate of clearance attesting to its low t_1/2_ of 2 h. Additionally, a single oral dose led to a sustained increase (>4 h) in plasma PTH levels, owing to its lipophilic nature which possibly causes prolonged systemic exposure (91, 92).

Despite harboring promise, calcilytics failed in treating osteoporosis in clinical settings; it has been well-reviewed but we will summarize the findings (93). Three calcilytic compounds, namely ronacaleret, JTT-305/MK-5442, and AXT914, were able to advance into Phase II clinical trials but halted due to lack of efficacy at pre-specified interim analyses. However, they were well-tolerated and had no off-target effects that staked their safety/efficacy (92, 93). Their failure due to on-target effects on bone is because administering calcilytics is equivalent to ablating the CaSR and blocking the CaSR in bone cells has been shown to influence bone turnover (93–95). There have been many studies to show the essential role of the CaSR in osteoblast differentiation, survival, and proliferation (96). In osteoclasts, calcilytics have a different pharmacological profile, as compared to parathyroid cells, which can be due to decreasing pH in the resorption pits which makes the CaSR less responsive (96–98).

### Calcilytics and Cancer

We previously stated that the CaSR acts as an oncogene in the prostrate, ovarian, and testicular cancer. In addition, Brenner lab showed that the bone metastasis caused by renal cell carcinoma (RCC) was promoted by the extracellular Ca^2+^ through the CaSR. They found that the CaSR was highly expressed in patient samples from those with bone metastases as compared to those with no or lung metastases. The calcilytic NPS-2143 acted in a predictable manner by blunting the response to calcium induced migration and proliferation of bone metastatic primary RCC cells (99). Very recently, their lab also implicated the receptor in promoting bone metastases in mice, using 786-O (RCC) cells with stably transfected *CaSR* gene. NPS-2143 was able to inhibit the phosphorylation of SHC, AKT, ERK, JNK and p90RSK in response to high Ca^2+^ in these transfected cells (100).

An overlooked aspect of the targeting CaSR is its interplay with cytokines and growth factors, which is quite interesting given that they play a significant role in cancer. Nielsen et al. were probably the first ones who demonstrated the positive correlation between the cytokine IL-1β and CaSR mRNA levels in bovine parathyroid gland samples. They were investigating the paracrine nature of IL-1 on PTH secretion, PTH mRNA, and CaSR mRNA; IL-1β (2,000 pg/ml) upregulated CaSR mRNA levels by 180% (of control) and an IL-1 receptor antagonist abolished this effect (101). The first direct evidence of the involvement of the endogenously expressed CaSR in the secretion of multiple cytokines and growth factors by metastatic breast cancer cells was reported by Hernández-Bedolla et al. They reported that CaSR activation in MDA-MB-231 cells transactivated EGFR and stimulated the secretion of endothelial chemotactic and pro-angiogenic factors like GM-CSF (granulocyte-macrophage colony stimulating factor), EGF (epidermal growth factor), MDC (macrophage-derived chemokine), FGF-4 (fibroblast growth factor-4), and IGFBP-2 (insulin-like growth factor-binding protein-2). The receptor was also shown to diminish the constitutive secretion IL-6 and β-NGF (β-nerve growth factor). It was interesting to see that common angiogenic factors (like Vascular endothelial growth factor) and pro-inflammatory cytokines (like TNF-a, IL-1a, IL-10, and others) were not detectable in response to PAE (porcine aortic endothelial) cells in the microarrays used for screening, implying that the CaSR is selectively responsible for promoting a specific set of growth factors and cytokines. As anticipated, NPS-2143 antagonized the receptor response, inhibited the secretion of the mentioned factors, and attenuated the angiogenic effect of the breast cancer cells on PAE cells (102). An apparent paradox regarding the secretory patterns of IL-6 was addressed later by the same lab. They observed that basal activity of the endogenously expressed receptor in MDA-MB-231 cells stimulated IL-6 secretion, but agonist stimulation seemingly had an opposite effect. Agonist stimulation, with 1.85 mM Ca^2+^ and R568 (a calcimimetic), engages the CaSR in Rab11a dependent endosomal recycling and PI3K signaling pathway, crucial in inhibiting IL-6 secretion. To compare it with “normal” mammary epithelial MCF-12A cells, CaSR stimulation increased IL-6 secretion. Regardless of cell type and receptor activation, the calcilytic NPS-2143 decreased detectable IL-6 levels in the cell culture supernatants (103). The estrogen receptor (ER) activity induced by high calcium levels in ER+ breast cancer cells were also evidenced to be partly rescued with calcilytics (88). A recent report on the CaSR promoting gastric cancer progression mentions a few experiments where they used calcilytics *in vivo* to bring down tumor growth and metastasis. Mice bearing xenografted tumors injected with CaCl_2_ or CaCl_2_ plus NPS-2143 at the site of implantation had around 46% reduction in tumor volume with the treatment. Also, MKN45 cells pre-cocultured with or without NPS-2143 had a significantly lesser number of metastatic tumors when injected intraperitoneally in nude mice (104). This is quite intriguing as it is the first time to our knowledge where calcilytics have been used *in vivo*, albeit intratumorally, to directly target tumors where CaSR aids cancer progression. The team went on to show that there is a functional link between CaSR and human telomerase reverse transcriptase (hTERT) in gastric cancers, where calcilytics inhibited Ca-mediated upregulation of hTERT and accumulation of p-Akt (105). A more direct effect of calcilytics on gastric cancer cells was also reported, where NPS-2143 inhibited migration, invasion, proliferation, and promoted apoptosis. They also reported the upregulation of CaSR in gastric cancer cells and tissues (106). A short communication from Yamamura et al. showed that the calcilytic NPS-2143 inhibited the proliferation and migration in prostate cancer cells, suggesting their therapeutic potential (107). In all these types of cancer where the CaSR is upregulated or acts as an oncogene, like in breast cancer, the effect of calcilytics in impeding metastasis is highlighted.

Every therapeutic decision involves a risk-benefit tradeoff. The CaSR might be therapeutically challenging to target due to its ubiquity and its role in maintaining physiological functions. Systemic administration of calcilytics may result in unpredictable effects in a complex disease like breast cancer. The main risk would be exacerbating existing hypercalcemia as data amassed from the various clinical trials with calcilytics showed pre-dose serum Ca^2+^ to be elevated in many cases (93). If calcilytics are suggested as a therapy, it would be important to diagnose if the patient has hypercalcemia and whether it arose from HHM with osseous involvement, or hyperparathyroidism from adenomas (108) because calcilytics would be detrimental in the latter case. Besides this, clinical trials attest that calcilytics were devoid of any other major side-effects (90).

Calcilytics were developed to treat osteoporosis by a bone-anabolic effect and mimics an intermittent PTH treatment. Continual exposure to PTH has been shown to have catabolic effect but intermittent administration of PTH or PTHrP results in net bone formation (109–111). Swami et al. showed that this effect of intermittent PTH treatment reduced cancer cell engraftment and incidence of skeletal tumors *in vivo* in various models involving MDA-MB-231 cells or 4T1 murine human breast cancer tumors, and that it rendered a less metastatic phenotype to the cells. However, pre-treatment of mice with intermittent PTH in an orthotopic 4T1 breast cancer model didn't affect the primary tumor volume. In other pre-treatment or treatment models, the treatment reduced skeletal metastases but didn't affect metastases to other internal organs (112). This effect of intermittent PTH treatment on the bone microenvironment to hinder metastases to bones can also be a mechanism through which calcilytics could function in reducing tumor burden. Whether or not calcilytics are able to release the desired amount of PTH to reach the aforementioned effect require further investigation. The lack of tissue selectivity of calcilytics is still a challenge and it needs further development to prevent off-target effects or on-target effects in the bone, but it is interesting that it can probably infiltrate the bone microenvironment. However, when coupled to 17β-estradiol in ovariectomized rats, it led to increase in cancellous bone area (97), which opens up an interesting possibility of coupling anti-resorptives and calcilytics.

## Therapeutic Perspective

**Figure 2:**
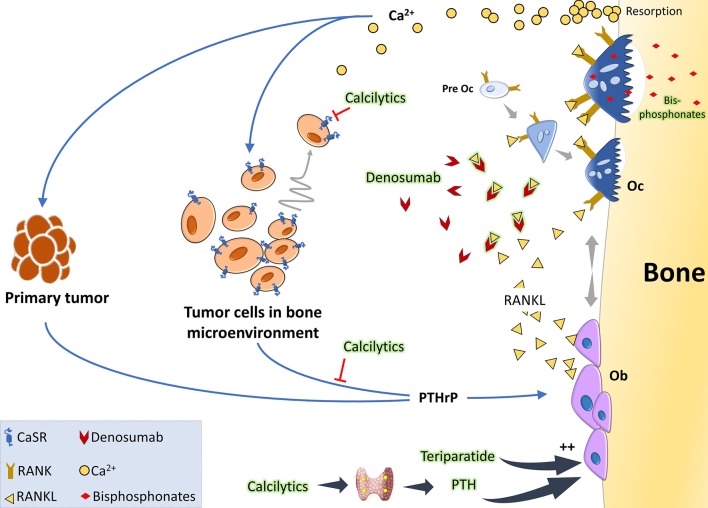
The vicious cycle of osteolytic metastasis in breast cancer and therapeutic targets: tumor cells produce PTHrP which acts on osteoblasts (Ob) to stimulate production of RANKL. When RANKL binds to its receptor RANK, osteoclast precursor cells undergo differentiation to activated osteoclasts (Oc). These activated Oc now undergo maturation and start active resorption which releases copious amount of Ca^2+^ and growth factors. Acting via the CaSR on the tumor cells, Ca^2+^ is considered to be a chemoattractant and also further stimulates PTHrP synthesis, thereby aggravating the vicious cycle. Clinically, the standard of care is either RANKL inhibitors like denosumab which binds to it and prevents it from interacting with its receptor RANK or bisphosphonates which are metabolic poisons taken up by the osteoclasts during resorption. Anabolic therapies, like intermittent teriparatide or the intermittent PTH effect mimicked by administering calcilytics, can be a promising option as they would increase Ob activity. Calcilytics, which are CaSR antagonists, can also aid by decreasing the chemoattraction of the tumor cells to the Ca^2+^ gradient and by hindering PTHrP production.

The principal therapeutic strategies in the market aim at targeting osteoclasts, given that most bone metastases intersect with exacerbated osteoclast activity (Figure 2). Under the broad umbrella of antiresorptive therapies, one major category is a class of compounds called bisphosphonates, which in essence are metabolic poisons inducing apoptosis in osteoclasts. The prodigal drug of this class appears to be zolendronate, which has braved various clinical trials to show its effectiveness in reducing skeletal related events (SREs) and also shown to have anticancer activity when used as an adjuvant or neoadjuvant (113). Unfortunately, this therapy comes with its share of side-effects like osteonecrosis of the jaw, bone pain, hypocalcemia, and fractures. Also their high affinity for the bone makes them build up in the tissues and causes adynamic bones (114, 115). Another major category is the humanized anti-RANKL antibody, denosumab, which acts by blocking the association of RANK-RANKL and in turn blocks osteoclastogenesis. Denosumab appears to be the preferred anti-resorptive therapy in the market as phase III clinical trials showed that they are more effective in delaying SREs compared to zolendronate (116). Side-effects of this therapy include hypocalcemia, nausea, fatigue, and osteonecrosis of the jaw (116, 117). Both these therapies have also highlighted their role in antitumoral effects by aiding the antitumor immune system (118, 119). In patients with osteolytic bone diseases who are put on such therapies, the disease often progresses and in 50% of the patients there is a recurrence with SREs (120). Recent alternative therapies for targeting osteoclasts include Cathepsin-K inhibitors, c-src inhibitors, and integrin inhibitors (121–123). Some cathepsin-K inhibitors made their way to clinical trials but had to be discontinued due their side effects on skin, risk of atrial fibrillation, and stroke. There are ongoing trials with c-src inhibitors like Dasatinib and Saracatinib, and also with anti-αvβ3 integrin MABs (Etaracizumab) (113, 115).

Since metastatic cancer cells are unable to destroy the bone on their own, they hijack the bone cells to create an environment favorable for their growth leading to the dire complications associated with bone metastases. Thus, it is quite understandable why most of the drugs in the market for bone metastatic patients are bone targeted. An interesting proposition about harnessing osteoblasts, based on data from patients, *in vivo* experiments, and co cultures, suggest that osteoblasts and their secretomes were hostile to growth of myeloma cells in the bone (124, 125). Similar *in vitro* data in the case of breast cancer also showed that introduction of osteoblasts curbed bone turnover caused by osteolytic breast cancer (126). Although other therapies have been tackling cancer related bone diseases, regaining bone health and quality remains a challenge. It wouldn't be far-fetched to talk about bone anabolic agents in such cases. The most widely used anabolic agent is teriparatide (PTH 1-34) for osteoporosis and was shown to suppress myeloma growth. The use of teriparatide in the clinic on patients with cancer is highly improbable as it was shown to increase incidences of osteosarcoma in rodents- but the anti-tumor effects of PTH warrants further investigation into the use of bone anabolic agents against osteolytic breast cancer. Calcilytics can be a strong contender as there is mounting evidence toward its inhibitory effects on metastasis of cancer cells as discussed and that it has a bone anabolic effect. The risk of using such compounds are that it may exacerbate the hypercalcemia, and by blocking renal excretion of Ca^2+^, further raise serum Ca^2+^ levels. Research on calcilytics for such indications should be aimed at increasing its tissue specificity, reducing off-target effects, targeted delivery, and finding out its interaction with current therapies in the market. A drug with direct actions on tumor cells and having a potent anabolic effect might be quite helpful in the clinic.

## Conclusion

Since the discovery and cloning of the extracellular CaSR, we have been able to throw light on its vital role in orchestrating calcium homeostasis in the body. However, among its non-calciotropic roles, it has been shown to be expressed in various organs like the breast and implicated in numerous cellular processes like differentiation, migration, proliferation, etc. During lactation, it coordinates maternal and neonatal calcium metabolism. However, in a diseased setting, where there is rising evidence of the CaSR acting as an oncogene in breast cancer, it is said to facilitate a vicious cycle of osteolysis and tumor growth affecting the pathophysiology of bone metastases. Breast cancer is the prime cause of cancer mortality in women and although we have come a long way in diagnosis, treatment, and disease management, metastatic disease remains a major challenge. Current therapies are bone-targeted, and we present a case where CaSR antagonists can be an alternate strategy or further improve the standard-of-care. The CaSR may sit in a cascade of complex signaling events and it would be worth investigating if CaSR based agents can prevent or delay bone destruction, even though more work is required to elucidate the intricacies of its role and for producing better targeted drugs.

## Author Contributions

SD and RM conceived the idea for the review. SD wrote the manuscript under the guidance of RM. PC, SK, and MB contributed to substantial inputs which helped structure the review and frame the concepts.

### Conflict of Interest

The authors declare that the research was conducted in the absence of any commercial or financial relationships that could be construed as a potential conflict of interest.

## References

[1] Nemeth EF. Misconceptions about calcimimetics. Ann N Y Acad Sci. (2006) 1068:471–6. 10.1196/annals.1346.044 16831944

[2] McLean FC Unsolved problems of parathyroid physiology. In: Greep RO Talmage RV, editors. The Parathyroids. Springfield, IL: Charles C. Thomas (1961). p. 7–18.

[3] López-Barneo J Armstrong CM. Depolarizing response of rat parathyroid cells to divalent cations. J Gen Physiol. (1983) 82:269–94. 10.1085/jgp.82.2.269 6619798 PMC2228694

[4] Shoback DM Chen TH Injection of poly(A)+ RNA from bovine parathyroid tissue into *Xenopus oocytes* confers sensitivity to extracellular calcium. J. Bone Miner. Res. (1991) 6:S135. 7511319 10.1002/jbmr.5650090219

[5] Racke FK Dubyak GR Nemeth EF Functional expression of the parathyroid cell calcium receptor in Xenopus oocytes. J. Bone Miner. Res. (1991) 6:S118. 10.1016/0014-5793(93)80390-g 8224151

[6] Brown EM Gamba G Riccardi D Lombardi M Butters R Kifor O Cloning and characterization of an extracellular Ca^2+^-sensing receptor from bovine parathyroid. Nature. (1993) 366:575–80. 10.1038/366575a0 8255296

[7] Pollak MR Brown EM Chou Y-HW Hebert SC Marx SJ Stelnmann B Mutations in the human Ca^2+^-sensing receptor gene cause familial hypocalciuric hypercalcemia and neonatal severe hyperparathyroidism. Cell. (1993) 75:1297–303. 10.1016/0092-8674(93)90617-Y 7916660

[8] Zhang C Zhang T Zou J Miller CL Gorkhali R Yang J-Y. Structural basis for regulation of human calcium-sensing receptor by magnesium ions and an unexpected tryptophan derivative co-agonist. Sci Adv. (2016) 2:e1600241. 10.1126/sciadv.1600241 27386547 PMC4928972

[9] Chakravarti B Chattopadhyay N Brown EM. Signaling through the extracellular Calcium-Sensing Receptor (CaSR). In: Shahidul IM, editors, Calcium Signaling. Dordrecht: Springer (2012). p. 103–42. 10.1007/978-94-007-2888-2_5 22453940

[10] Nemeth EF. Regulation of cytosolic calcium by extracellular divalent cations in C-cells and parathyroid cells. Cell Calcium. (1990) 11:323–7. 10.1016/0143-4160(90)90033-Q 2194658

[11] Quinn SJ Ye CP Diaz R Kifor O Bai M Vassilev P. The Ca^2+^-sensing receptor: a target for polyamines. Am J Physiol. (1997) 273:C1315–23. 10.1152/ajpcell.1997.273.4.C1315 9357776

[12] Brown EM Katz C Butters R Kifor O. Polyarginine, polylysine, and protamine mimic the effects of high extracellular calcium concentrations on dispersed bovine parathyroid cells. J Bone Miner Res. (1991) 6:1217–25. 10.1002/jbmr.5650061112 1666808

[13] Ye C Ho-Pao CL Kanazirska M Quinn S Rogers K Seidman CE. Amyloid-beta proteins activate Ca(2+)-permeable channels through calcium-sensing receptors. J Neurosci Res. (1997) 47:547–54. 10.1002/(SICI)1097-4547(19970301)47:5<547::AID-JNR10>3.0.CO;2-V 9067864

[14] Brown EM Butters R Katz C Kifor O. Neomycin mimics the effects of high extracellular calcium concentrations on parathyroid function in dispersed bovine parathyroid cells. Endocrinology. (1991) 128:3047–54. 10.1210/endo-128-6-3047 1645260

[15] Brown E Enyedi P LeBoff M Rotberg J Preston J Chen C. High extracellular Ca^2+^ and Mg2+ stimulate accumulation of inositol phosphates in bovine parathyroid cells. FEBS Lett. (1987) 218:113–8. 10.1016/0014-5793(87)81029-3 3109945

[16] Wettschureck N Lee E Libutti SK Offermanns S Robey PG Spiegel AM. Parathyroid-specific double knockout of Gq and G11 alpha-subunits leads to a phenotype resembling germline knockout of the extracellular Ca^2+^ -sensing receptor. Mol Endocrinol. (2007) 21:274–80. 10.1210/me.2006-0110 16988000

[17] Hannan FM Babinsky VN Thakker RV. Disorders of the calcium-sensing receptor and partner proteins: insights into the molecular basis of calcium homeostasis. J Mol Endocrinol. (2016) 57:R127–42. 10.1530/JME-16-0124 27647839 PMC5064759

[18] Brennan T Rybchyn M Green W Atwa S Conigrave A Mason R. Osteoblasts play key roles in the mechanisms of action of strontium ranelate. Br J Pharmacol. (2009) 157:1291–300. 10.1111/j.1476-5381.2009.00305.x 19563530 PMC2743848

[19] Rybchyn MS Slater M Conigrave AD Mason RS. An Akt-dependent increase in canonical Wnt signaling and a decrease in sclerostin protein levels are involved in strontium ranelate-induced osteogenic effects in human osteoblasts. J Biol Chem. (2011) 286:23771–9. 10.1074/jbc.M111.251116 21566129 PMC3129158

[20] Worzfeld T Wettschureck N Offermanns S G12/G13-mediated signalling in mammalian physiology and disease. Trends Pharmacol Sci. (2008) 29:582–9. 10.1016/j.tips.2008.08.002 18814923

[21] Ward DT Riccardi D. New concepts in calcium-sensing receptor pharmacology and signalling. Br J Pharmacol. (2012) 165:35–48. 10.1111/j.1476-5381.2011.01511.x 21627634 PMC3252964

[22] Brennan SC Thiem U Roth S Aggarwal A Fetahu ISh Tennakoon S. Calcium sensing receptor signalling in physiology and cancer. Biochim Biophys Acta. (2013) 1833:1732–44. 10.1016/j.bbamcr.2012.12.011 23267858

[23] Mamillapalli R Wysolmerski J. The calcium-sensing receptor couples to Gαs and regulates PTHrP and ACTH secretion in pituitary cells. J Endocrinol. (2010) 204:287–97. 10.1677/JOE-09-0183 20032198 PMC3777408

[24] Brennan SC Wilkinson WJ Tseng H-E Finney B Monk B Dibble H. The extracellular calcium-sensing receptor regulates human fetal lung development via CFTR. Sci Rep. (2016) 6. 10.1038/srep21975 26911344 PMC4766410

[25] Conigrave AD Ward DT. Calcium-sensing receptor (CaSR): pharmacological properties and signaling pathways. Best Pract Res Clin Endocrinol Metab. (2013) 27:315–31. 10.1016/j.beem.2013.05.010 23856262

[26] Thomsen ARB Smajilovic S Bräuner-Osborne H. Novel strategies in drug discovery of the calcium-sensing receptor based on biased signaling. Curr Drug Targets. (2012) 13:1324–35. 10.2174/138945012802429642 22702634

[27] Leach K Conigrave AD Sexton PM Christopoulos A. Towards tissue-specific pharmacology: insights from the calcium-sensing receptor as a paradigm for GPCR (patho)physiological bias. Trends Pharmacol Sci. (2015) 36:215–25. 10.1016/j.tips.2015.02.004 25765207

[28] Hofer AM Brown EM. Calcium: extracellular calcium sensing and signalling. Nat Rev Mol Cell Biol. (2003) 4:530–8. 10.1038/nrm1154 12838336

[29] Pollak MR Brown EM Estep HL McLaine PN Kifor O Park J Autosomal dominant hypocalcaemia caused by a Ca^2+^-sensing receptor gene mutation. Nat Genet. (1994) 8:303–7. 10.1038/ng1194-303 7874174

[30] Watanabe S Fukumoto S Chang H Takeuchi Y Hasegawa Y Okazaki R. Association between activating mutations of calcium-sensing receptor and Bartter's syndrome. Lancet. (2002) 360:692–4. 10.1016/S0140-6736(02)09842-2 12241879

[31] Alfadda TI Saleh AMA Houillier P Geibel JP. Calcium-sensing receptor 20 years later. Am J Physiol Cell Physiol. (2014) 307:C221–31. 10.1152/ajpcell.00139.2014 24871857 PMC4121584

[32] Aida K Koishi S Tawata M Onaya T. Molecular cloning of a putative Ca(2+)-sensing receptor cDNA from human kidney. Biochem Biophys Res Commun. (1995) 214:524–9. 10.1006/bbrc.1995.2318 7677761

[33] Ray JM Squires PE Curtis SB Meloche MR Buchan AM. Expression of the calcium-sensing receptor on human antral gastrin cells in culture. J Clin Invest. (1997) 99:2328–33. 10.1172/JCI119413 9153273 PMC508070

[34] Chattopadhyay N Cheng I Rogers K Riccardi D Hall A Diaz R. Identification and localization of extracellular Ca(2+)-sensing receptor in rat intestine. Am J Physiol. (1998) 274:G122–30. 10.1152/ajpgi.1998.274.1.G122 9458781

[35] Bikle DD Ratnam A Mauro T Harris J Pillai S. Changes in calcium responsiveness and handling during keratinocyte differentiation. Potential role of the calcium receptor. J Clin Invest. (1996) 97:1085–93. 10.1172/JCI118501 8613532 PMC507156

[36] Tu C-L Chang W Xie Z Bikle DD. Inactivation of the calcium sensing receptor inhibits E-cadherin-mediated cell-cell adhesion and calcium-induced differentiation in human epidermal keratinocytes. J Biol Chem. (2008) 283:3519–28. 10.1074/jbc.M708318200 18065418

[37] Ruat M Molliver ME Snowman AM Snyder SH. Calcium sensing receptor: molecular cloning in rat and localization to nerve terminals. Proc Natl Acad Sci USA. (1995) 92:3161–5. 10.1073/pnas.92.8.3161 7724534 PMC42125

[38] Canaff L Petit JL Kisiel M Watson PH Gascon-Barré M Hendy GN. Extracellular calcium-sensing receptor is expressed in rat hepatocytes. coupling to intracellular calcium mobilization and stimulation of bile flow. J Biol Chem. (2001) 276:4070–9. 10.1074/jbc.M009317200 11071898

[39] Tfelt-Hansen J Hansen JL Smajilovic S Terwilliger EF Haunso S Sheikh SP. Calcium receptor is functionally expressed in rat neonatal ventricular cardiomyocytes. Am J Physiol Heart Circ Physiol. (2006) 290:H1165–71. 10.1152/ajpheart.00821.2005 16243911

[40] Cheng I Klingensmith ME Chattopadhyay N Kifor O Butters RR Soybel DI. Identification and localization of the extracellular calcium-sensing receptor in human breast. J Clin Endocrinol Metab. (1998) 83:703–7. 10.1210/jcem.83.2.4558 9467597

[41] VanHouten J Dann P McGeoch G Brown EM Krapcho K Neville M. The calcium-sensing receptor regulates mammary gland parathyroid hormone–related protein production and calcium transport. J Clin Invest. (2004) 113:598–608. 10.1172/JCI200418776 14966569 PMC338258

[42] VanHouten JN Neville MC Wysolmerski JJ. The calcium-sensing receptor regulates plasma membrane calcium adenosine triphosphatase isoform 2 activity in mammary epithelial cells: a mechanism for calcium-regulated calcium transport into milk. Endocrinology. (2007) 148:5943–54. 10.1210/en.2007-0850 17823248 PMC7108505

[43] Kim W Wysolmerski JJ. Calcium-sensing receptor in breast physiology and cancer. Front Physiol. (2016) 7:440. 10.3389/fphys.2016.00440 27746743 PMC5043011

[44] Mamillapalli R VanHouten J Dann P Bikle D Chang W Brown E. Mammary-specific ablation of the calcium-sensing receptor during lactation alters maternal calcium metabolism, milk calcium transport, and neonatal calcium accrual. Endocrinology. (2013) 154:3031–42. 10.1210/en.2012-2195 23782944 PMC3749485

[45] Broadus AE Mangin M Ikeda K Insogna KL Weir EC Burtis WJ. Humoral hypercalcemia of cancer. Identification of a novel parathyroid hormone-like peptide. N Engl J Med. (1988) 319:556–63. 10.1056/NEJM198809013190906 3043221

[46] Hiremath M Wysolmerski J Role of PTHrP in mammary gland development and breast cancer. Clin Rev Bone Miner Metab. (2014) 12:178–89. 10.1007/s12018-014-9170-9

[47] Jüppner H Abou-Samra AB Freeman M Kong XF Schipani E Richards J. A G protein-linked receptor for parathyroid hormone and parathyroid hormone-related peptide. Science. (1991) 254:1024–6. 10.1126/science.1658941 1658941

[48] Sanders JL Chattopadhyay N Kifor O Yamaguchi T Butters RR Brown EM. Extracellular calcium-sensing receptor expression and its potential role in regulating parathyroid hormone-related peptide secretion in human breast cancer cell lines. Endocrinology. (2000) 141:4357–64. 10.1210/endo.141.12.7849 11108243

[49] Casalà C Gil-Guiñón E Ordóñez JL Miguel-Queralt S Rodríguez E Galván P. The calcium-sensing receptor is silenced by genetic and epigenetic mechanisms in unfavorable neuroblastomas and its reactivation induces ERK1/2-dependent apoptosis. Carcinogenesis. (2013) 34:268–76. 10.1093/carcin/bgs338 23108190

[50] Haven CJ van Puijenbroek M Karperien M Fleuren G-J Morreau H. Differential expression of the calcium sensing receptor and combined loss of chromosomes 1q and 11q in parathyroid carcinoma. J Pathol. (2004) 202:86–94. 10.1002/path.1489 14694525

[51] Fetahu IS Höbaus J Aggarwal A Hummel DM Tennakoon S Mesteri I. Calcium-sensing receptor silencing in colorectal cancer is associated with promoter hypermethylation and loss of acetylation on histone 3. Int J Cancer. (2014) 135:2014–23. 10.1002/ijc.28856 24691920 PMC4282356

[52] Hobson SA Wright J Lee F McNeil SE Bilderback T Rodland KD. Activation of the MAP kinase cascade by exogenous calcium-sensing receptor. Mol Cell Endocrinol. (2003) 200:189–98. 10.1016/S0303-7207(01)00749-3 12644311

[53] Feng J Xu X Li B Brown E Farris AB Sun S-Y. Prostate cancer metastatic to bone has higher expression of the calcium-sensing receptor (CaSR) than primary prostate cancer. Receptors Clin Investig. (2014) 1:e270. 10.14800/rci.270 26065011 PMC4459757

[54] Tfelt-Hansen J Schwarz P Terwilliger EF Brown EM Chattopadhyay N. Calcium-sensing receptor induces messenger ribonucleic acid of human securin, pituitary tumor transforming gene, in rat testicular cancer. Endocrinology. (2003) 144:5188–93. 10.1210/en.2003-0520 12970167

[55] Campos-Verdes LM Costa-Silva DR Silva-Sampaio JP da Barros-Oliveira M da C Escórcio-Dourado CS Martins LM. Review of polymorphism of the calcium-sensing receptor gene and breast cancer risk. Cancer Invest. (2018) 36:1–7. 10.1080/07357907.2018.1430817 29504802

[56] Yao S Haddad SA Hu Q Liu S Lunetta KL Ruiz-Narvaez EA. Genetic variations in vitamin D-related pathways and breast cancer risk in African American women in the AMBER consortium. Int J Cancer. (2016) 138:2118–26. 10.1002/ijc.29954 26650177 PMC5087916

[57] Li X Kong X Jiang L Ma T Yan S Yuan C. A genetic polymorphism (rs17251221) in the calcium-sensing receptor is associated with breast cancer susceptibility and prognosis. Cell Physiol Biochem. (2014) 33:165–72. 10.1159/000356659 24481145

[58] Yan S Yuan C Yang Q Li X Yang N Liu X. A genetic polymorphism (rs17251221) in the calcium-sensing receptor is associated with ovarian cancer susceptibility. Oncol Rep. (2015) 34:2151–5. 10.3892/or.2015.4179 26252839

[59] Wang L Widatalla SE Whalen DS Ochieng J Sakwe AM. Association of calcium sensing receptor polymorphisms at rs1801725 with circulating calcium in breast cancer patients. BMC Cancer. (2017) 17:511. 10.1186/s12885-017-3502-3 28764683 PMC5540567

[60] Promkan M Liu G Patmasiriwat P Chakrabarty S. BRCA1 suppresses the expression of survivin and promotes sensitivity to paclitaxel through the calcium sensing receptor (CaSR) in human breast cancer cells. Cell Calcium. (2011) 49:79–88. 10.1016/j.ceca.2011.01.003 21296416

[61] Mamillapalli R VanHouten J Zawalich W Wysolmerski J. Switching of G-protein usage by the calcium-sensing receptor reverses its effect on parathyroid hormone-related protein secretion in normal versus malignant breast cells. J Biol Chem. (2008) 283:24435–47. 10.1074/jbc.M801738200 18621740 PMC2528989

[62] Chilco PJ Leopold V Zajac JD. Differential regulation of the parathyroid hormone-related protein gene P1 and P3 promoters by cAMP. Mol Cell Endocrinol. (1998) 138:173–84. 10.1016/S0303-7207(97)00239-6 9685226

[63] Takagaki K Takashima T Onoda N Tezuka K Noda E Kawajiri H. Parathyroid hormone-related protein expression, in combination with nodal status, predicts bone metastasis and prognosis of breast cancer patients. Exp Ther Med. (2012) 3:963–8. 10.3892/etm.2012.521 22970000 PMC3438629

[64] Henderson MA Danks JA Slavin JL Byrnes GB Choong PFM Spillane JB. Parathyroid hormone-related protein localization in breast cancers predict improved prognosis. Cancer Res. (2006) 66:2250–6. 10.1158/0008-5472.CAN-05-2814 16489028

[65] Ghoussaini M Fletcher O Michailidou K Turnbull C Schmidt MK Dicks E. Genome-wide association analysis identifies three new breast cancer susceptibility loci. Nat Genet. (2012) 44:312–8. 10.1038/ng.1049 22267197 PMC3653403

[66] Kim W Takyar FM Swan K Jeong J VanHouten J Sullivan C. Calcium-sensing receptor promotes breast cancer by stimulating intracrine actions of parathyroid hormone–related protein. Cancer Res. (2016) 76:5348–60. 10.1158/0008-5472.CAN-15-2614 27450451 PMC5026591

[67] Yang Y Wang B. PTH1R-CaSR cross talk: new treatment options for breast cancer osteolytic bone metastases. Int J Endocrinol. (2018) 2018:8. 10.1155/2018/7120979 30151009 PMC6087585

[68] Colloton M Shatzen E Wiemann B Starnes C Scully S Henley C. Cinacalcet attenuates hypercalcemia observed in mice bearing either Rice H-500 Leydig cell or C26-DCT colon tumors. Eur J Pharmacol. (2013) 712:8–15. 10.1016/j.ejphar.2013.04.013 23623934

[69] Paget S. The distribution of secondary growths in cancer of the breast. 1889. Cancer Metastasis Rev. (1989) 8:98–101. 2673568

[70] Guise T. Examining the metastatic niche: targeting the microenvironment. Semin Oncol. (2010) 37(Suppl. 2):S2–14. 10.1053/j.seminoncol.2010.10.007 21111245

[71] Saidak Z Boudot C Abdoune R Petit L Brazier M Mentaverri R. Extracellular calcium promotes the migration of breast cancer cells through the activation of the calcium sensing receptor. Exp Cell Res. (2009) 315:2072–80. 10.1016/j.yexcr.2009.03.003 19285978

[72] Boudot C Hénaut L Thiem U Geraci S Galante M Saldanha P. Overexpression of a functional calcium-sensing receptor dramatically increases osteolytic potential of MDA-MB-231 cells in a mouse model of bone metastasis through epiregulin-mediated osteoprotegerin downregulation. Oncotarget. (2017) 8:56460. 10.18632/oncotarget.16999 28915604 PMC5593575

[73] Tharmalingam S Daulat AM Antflick JE Ahmed SM Nemeth EF Angers S. Calcium-sensing receptor modulates cell adhesion and migration via integrins. J Biol Chem. (2011) 286:40922–33. 10.1074/jbc.M111.265454 21969374 PMC3220485

[74] Saidak Z Mentaverri R Brown EM. The role of the calcium-sensing receptor in the development and progression of cancer. Endocr Rev. (2009) 30:178–95. 10.1210/er.2008-0041 19237714

[75] Lam BS Cunningham C Adams GB. Pharmacologic modulation of the calcium-sensing receptor enhances hematopoietic stem cell lodgment in the adult bone marrow. Blood. (2011) 117:1167–75. 10.1182/blood-2010-05-286294 21076044 PMC3056470

[76] Morgan MP Cooke MM McCarthy GM. Microcalcifications associated with breast cancer: an epiphenomenon or biologically significant feature of selected tumors? J Mammary Gland Biol Neoplasia. (2005) 10:181–7. 10.1007/s10911-005-5400-6 16025224

[77] Rominger MB Steinmetz C Westerman R Ramaswamy A Albert U-S. microcalcification-associated breast cancer: presentation, successful first excision, long-term recurrence and survival rate. Breast Care. (2015) 10:380–5. 10.1159/000440794 26989356 PMC4789874

[78] Bellahcène A Castronovo V. Expression of bone matrix proteins in human breast cancer: potential roles in microcalcification formation and in the genesis of bone metastases. Bull Cancer. (1997) 84:17–24. 9180854

[79] Wilkinson L Thomas V Sharma N. Microcalcification on mammography: approaches to interpretation and biopsy. Br J Radiol. (2019) 90:2016094. 10.1259/bjr.20160594 27648482 PMC5605030

[80] Cox RF Jenkinson A Pohl K O'Brien FJ Morgan MP. Osteomimicry of mammary adenocarcinoma cells *in vitro*; increased expression of bone matrix proteins and proliferation within a 3D collagen environment. PLoS ONE. (2012) 7:e41679. 10.1371/journal.pone.0041679 22911843 PMC3404045

[81] Davis FM Azimi I Faville RA Peters AA Jalink K Putney JW. Induction of epithelial-mesenchymal transition (EMT) in breast cancer cells is calcium signal dependent. Oncogene. (2014) 33:2307–16. 10.1038/onc.2013.187 23686305 PMC3917976

[82] El Hiani Y Ahidouch A Lehen'kyi V Hague F Gouilleux F Mentaverri R. Extracellular signal-regulated kinases 1 and 2 and TRPC1 channels are required for calcium-sensing receptor-stimulated MCF-7 breast cancer cell proliferation. Cell Physiol Biochem. (2009) 23:335–46. 10.1159/000218179 19471101

[83] El Hiani Y Lehen'kyi V Ouadid-Ahidouch H Ahidouch A. Activation of the calcium-sensing receptor by high calcium induced breast cancer cell proliferation and TRPC1 cation channel over-expression potentially through EGFR pathways. Arch Biochem Biophys. (2009) 486:58–63. 10.1016/j.abb.2009.03.010 19332022

[84] Aggarwal A Prinz-Wohlgenannt M Gröschel C Tennakoon S Meshcheryakova A Chang W. The calcium-sensing receptor suppresses epithelial-to-mesenchymal transition and stem cell- like phenotype in the colon. Molecular Cancer. (2015) 14:61. 10.1186/s12943-015-0330-4 25879211 PMC4405849

[85] Singh N Aslam MN Varani J Chakrabarty S. Induction of calcium sensing receptor in human colon cancer cells by calcium, vitamin D and aquamin: promotion of a more differentiated, less malignant and indolent phenotype. Mol Carcinogen. (2015) 54:543–53. 10.1002/mc.22123 26076051

[86] Wen L Sun L Xi Y Chen X Xing Y Sun W. Expression of calcium sensing receptor and E-cadherin correlated with survival of lung adenocarcinoma. Thoracic Cancer. (2015) 6:754–60. 10.1111/1759-7714.12255 26557914 PMC4632928

[87] Hua H Zhang H Kong Q Jiang Y. Mechanisms for estrogen receptor expression in human cancer. Exp Hematol Oncol. (2018) 7:24. 10.1186/s40164-018-0116-7 30250760 PMC6148803

[88] Journé F Dumon J-C Kheddoumi N Fox J Laios I Leclercq G. Extracellular calcium downregulates estrogen receptor alpha and increases its transcriptional activity through calcium-sensing receptor in breast cancer cells. Bone. (2004) 35:479–88. 10.1016/j.bone.2004.03.021 15268900

[89] Mentaverri R Yano S Chattopadhyay N Petit L Kifor O Kamel S. The calcium sensing receptor is directly involved in both osteoclast differentiation and apoptosis. FASEB J. (2006) 20:2562–4. 10.1096/fj.06-6304fje 17077282

[90] Nemeth EF Anabolic therapy for osteoporosis: calcilytics. IBMS BoneKEy. (2008) 5:196–208. 10.1138/20080318

[91] Nemeth EF Delmar EG Heaton WL Miller MA Lambert LD Conklin RL. Calcilytic compounds: potent and selective Ca^2+^receptor antagonists that stimulate secretion of parathyroid hormone. J Pharmacol Exp Ther. (2001) 299:323−31. 11561095

[92] Nemeth EF Van Wagenen BC Balandrin MF Chapter one - discovery and development of calcimimetic and calcilytic compounds. In: Witty DR Cox B, editors. Progress in Medicinal Chemistry. Elsevier (2018). p. 1–86. 10.1016/bs.pmch.2017.12.001 29680147

[93] Nemeth EF Goodman WG. Calcimimetic and calcilytic drugs: feats, flops, and futures. Calcif Tissue Int. (2016) 98:341–58. 10.1007/s00223-015-0052-z 26319799

[94] Dvorak MM Siddiqua A Ward DT Carter DH Dallas SL Nemeth EF. Physiological changes in extracellular calcium concentration directly control osteoblast function in the absence of calciotropic hormones. Proc Natl Acad Sci USA. (2004) 101:5140–5. 10.1073/pnas.0306141101 15051872 PMC387387

[95] Goltzman D Hendy GN. The calcium-sensing receptor in bone—mechanistic and therapeutic insights. Nat Rev Endocrinol. (2015) 11:298–307. 10.1038/nrendo.2015.30 25752283

[96] Santa Maria C Cheng Z Li A Wang J Shoback D Tu C-L. Interplay between CaSR and PTH1R signaling in skeletal development and osteoanabolism. Semin Cell Dev Biol. (2016) 49:11–23. 10.1016/j.semcdb.2015.12.004 26688334 PMC4761456

[97] Gowen M Stroup GB Dodds RA James IE Votta BJ Smith BR. Antagonizing the parathyroid calcium receptor stimulates parathyroid hormone secretion and bone formation in osteopenic rats. J Clin Invest. (2000) 105:1595–604. 10.1172/JCI9038 10841518 PMC300853

[98] Quinn SJ Bai M Brown EM. pH sensing by the calcium-sensing receptor. J Biol Chem. (2004) 279:37241–9. 10.1074/jbc.M404520200 15201280

[99] Joeckel E Haber T Prawitt D Junker K Hampel C Thüroff JW. High calcium concentration in bones promotes bone metastasis in renal cell carcinomas expressing calcium-sensing receptor. Molecular Cancer. (2014) 13:42. 10.1186/1476-4598-13-42 24576174 PMC3945739

[100] Frees S Breuksch I Haber T Bauer H-K Chavez-Munoz C Raven P. Calcium-sensing receptor (CaSR) promotes development of bone metastasis in renal cell carcinoma. Oncotarget. (2018) 9:15766–79. 10.18632/oncotarget.24607 29644008 PMC5884663

[101] Nielsen PK Rasmussen AK Butters R Feldt-Rasmussen U Bendtzen K Diaz R. Inhibition of PTH secretion by interleukin-1 beta in bovine parathyroid glands *in vitro* is associated with an up-regulation of the calcium-sensing receptor mRNA. Biochem Biophys Res Commun. (1997) 238:880–5. 10.1006/bbrc.1997.7207 9325185

[102] Hernández-Bedolla MA Carretero-Ortega J Valadez-Sánchez M Vázquez-Prado J Reyes-Cruz G. Chemotactic and proangiogenic role of calcium sensing receptor is linked to secretion of multiple cytokines and growth factors in breast cancer MDA-MB-231 cells. Biochim Biophys Acta. (2015) 1853:166–82. 10.1016/j.bbamcr.2014.10.011 25409930

[103] Hernández-Bedolla MA González-Domínguez E Zavala-Barrera C Gutiérrez-López TY Hidalgo-Moyle JJ Vázquez-Prado J. Calcium-sensing-receptor (CaSR) controls IL-6 secretion in metastatic breast cancer MDA-MB-231 cells by a dual mechanism revealed by agonist and inverse-agonist modulators. Mol Cell Endocrinol. (2016) 436:159–68. 10.1016/j.mce.2016.07.038 27477783

[104] Xie R Xu J Xiao Y Wu J Wan H Tang B. Calcium promotes human gastric cancer via a novel coupling of calcium-sensing receptor and TRPV4 channel. Cancer Res. (2017) 77:6499–512. 10.1158/0008-5472.CAN-17-0360 28951460

[105] Xie R Tuo B Yang S Chen X-Q Xu J. Calcium-sensing receptor bridges calcium and telomerase reverse transcriptase in gastric cancers via Akt. Clin Transl Oncol. (2019). 10.1007/s12094-019-02226-4 31650467

[106] Zhang Z-L Li Z-R Li J-S Wang S-R. Calcium-sensing receptor antagonist NPS-2143 suppresses proliferation and invasion of gastric cancer cells. Cancer Gene Ther. (2019). 10.1038/s41417-019-0128-4 31391530

[107] Yamamura A Nayeem MJ Sato M. Calcilytics inhibit the proliferation and migration of human prostate cancer PC-3 cells. J Pharmacol Sci. (2019) 139:254–7. 10.1016/j.jphs.2019.01.008 30808588

[108] Sigvaldason H Obayan A von Kuster K Pathak KA. Hypercalcemia in metastatic breast cancer unrelated to skeletal metastasis. CMAJ. (2016) 188:E91–4. 10.1503/cmaj.150638 26504099 PMC4786403

[109] Horwitz MJ Tedesco MB Sereika SM Prebehala L Gundberg CM Hollis BW. A 7-day continuous infusion of PTH or PTHrP suppresses bone formation and uncouples bone turnover. J Bone Miner Res. (2011) 26:2287–97. 10.1002/jbmr.415 21544866 PMC3304443

[110] Neer RM Arnaud CD Zanchetta JR Prince R Gaich GA Reginster JY. Effect of parathyroid hormone (1–34) on fractures and bone mineral density in postmenopausal women with osteoporosis. N Engl J Med. (2001) 344:1434–41. 10.3171/foc.2001.11.2.8 11346808

[111] Miller PD Hattersley G Riis BJ Williams GC Lau E Russo LA. Effect of abaloparatide vs. placebo on new vertebral fractures in postmenopausal women with osteoporosis: a randomized clinical trial. JAMA. (2016) 316:722–33. 10.1001/jama.2016.11136 27533157

[112] Swami S Johnson J Bettinson LA Kimura T Zhu H Albertelli MA. Prevention of breast cancer skeletal metastases with parathyroid hormone. JCI Insight. (2017) 2:90874. 10.1172/jci.insight.90874 28878134 PMC5621896

[113] Sousa S Clézardin P. Bone-targeted therapies in cancer-induced bone disease. Calcif Tissue Int. (2018) 102:227–50. 10.1007/s00223-017-0353-5 29079995

[114] Reyes C Hitz M Prieto-Alhambra D Abrahamsen B. Risks and benefits of bisphosphonate therapies. J Cell Biochem. (2016) 117:20–8. 10.1002/jcb.25266 26096687

[115] Maurizi A Rucci N. The osteoclast in bone metastasis: player and target. Cancers. (2018) 10:218. 10.3390/cancers10070218 29954079 PMC6071064

[116] Rosen LS Gordon D Tchekmedyian S Yanagihara R Hirsh V Krzakowski M. Zoledronic acid versus placebo in the treatment of skeletal metastases in patients with lung cancer and other solid tumors: a phase III, double-blind, randomized trial–the Zoledronic Acid Lung Cancer and Other Solid Tumors Study Group. J Clin Oncol. (2003) 21:3150–7. 10.1200/JCO.2003.04.105 12915606

[117] Body J-J Bone HG de Boer RH Stopeck A Van Poznak C Damião R. Hypocalcaemia in patients with metastatic bone disease treated with denosumab. Eur J Cancer. (2015) 51:1812–21. 10.1016/j.ejca.2015.05.016 26093811

[118] Lüftner D Niepel D Steger GG. Therapeutic approaches for protecting bone health in patients with breast cancer. Breast. (2018) 37:28–35. 10.1016/j.breast.2017.10.007 29073497

[119] Zekri J Mansour M Karim SM. The anti-tumour effects of zoledronic acid. J Bone Oncol. (2014) 3:25–35. 10.1016/j.jbo.2013.12.001 26909294 PMC4723416

[120] Coleman RE Major P Lipton A Brown JE Lee K-A Smith M. Predictive value of bone resorption and formation markers in cancer patients with bone metastases receiving the bisphosphonate zoledronic acid. J Clin Oncol. (2016) 23:4925–35. 10.1200/JCO.2005.06.091 15983391

[121] Jensen AB Wynne C Ramirez G He W Song Y Berd Y. The cathepsin K inhibitor odanacatib suppresses bone resorption in women with breast cancer and established bone metastases: results of a 4-week, double-blind, randomized, controlled trial. Clin Breast Cancer. (2010) 10:452–8. 10.3816/CBC.2010.n.059 21147688

[122] Yang JC Bai L Yap S Gao AC Kung H-J Evans CP. Effect of the specific Src family kinase inhibitor saracatinib on osteolytic lesions using the PC-3 bone model. Mol Cancer Ther. (2010) 9:1629–37. 10.1158/1535-7163.MCT-09-1058 20484016

[123] Carter RZ Micocci KC Natoli A Redvers RP Paquet-Fifield S Martin ACBM. Tumour but not stromal expression of β3 integrin is essential, and is required early, for spontaneous dissemination of bone-metastatic breast cancer. J Pathol. (2015) 235:760–72. 10.1002/path.4490 25430721

[124] Yaccoby S Wezeman MJ Zangari M Walker R Cottler-Fox M Gaddy D. Inhibitory effects of osteoblasts and increased bone formation on myeloma in novel culture systems and a myelomatous mouse model. Haematologica. (2006)91:192–9. 16461303 PMC1592551

[125] Adamik J Galson DL Roodman GD. Osteoblast suppression in multiple myeloma bone disease. J Bone Oncol. (2018) 13:62–70. 10.1016/j.jbo.2018.09.001 30591859 PMC6303385

[126] Krawetz R Wu YE Rancourt DE Matyas J. Osteoblasts suppress high bone turnover caused by osteolytic breast cancer *in vitro*. Exp Cell Res. (2009) 315:2333–42. 10.1016/j.yexcr.2009.04.026 19433087

